# Depressive symptoms and hypoglycaemic risk in individuals with type 2 diabetes mellitus: insights from the ACCORD-HRQL study

**DOI:** 10.1192/bjo.2025.10853

**Published:** 2025-10-13

**Authors:** Wanying Hong, Yang Yang, Zhenhua Xing

**Affiliations:** Department of Anaesthesiology, Hunan Provincial People’s Hospital (First Affiliated Hospital of Hunan Normal University), Changsha, China; Department of Cardiovascular Medicine, The Second Xiangya Hospital, Central South University, Changsha, China; Department of Emergency Medicine, Second Xiangya Hospital, Central South University, Changsha, China

**Keywords:** Type 2 diabetes mellitus, depression, hypoglycemia, nine-item PatientHealth Questionnaire

## Abstract

**Background:**

Depression in individuals with type 2 diabetes mellitus (T2DM) is associated with worse clinical prognosis; however, evidence regarding the relationship between depression and hypoglycaemic risk remains limited and inconclusive.

**Aim:**

Our study aimed to evaluate the association between depressive symptoms and hypoglycaemic events.

**Method:**

Depressive symptoms were assessed in participants of the ACCORD-HRQL study at baseline and during follow-up visits at 12, 36 and 48 months using the nine-item Patient Health Questionnaire (PHQ-9). Symptom severity was categorised into three levels: none (0–4 points), mild (5–9 points) or moderate to severe (10–24 points). The primary outcomes included hypoglycaemia requiring any assistance (HAA) and hypoglycaemia requiring medical assistance (HMA).

**Results:**

Over a median follow-up of 4.3 years, 220 individuals developed HAA (incidence rate: 27.0 per 1000 person-years) and 157 individuals experienced HMA (incidence rate: 18.8 per 1000 person-years). Depressive symptoms exhibited dynamic fluctuations during the study period, and participants with depression consistently demonstrated less effective glycaemic control compared to those without depression. However, each one-unit increase in PHQ-9 score was not associated with elevated risks of HAA (hazard ratio, 1.00; 95% CI, 0.97–1.03) or HMA (hazard ratio, 0.98; 95% CI, 0.95–1.02).

**Conclusions:**

Depressive symptoms in individuals with T2DM are dynamic and correlate with suboptimal glycaemic control. However, no significant association was observed between depression severity and increased hypoglycaemic events. These findings highlight the importance of integrated clinical strategies for continuous mental health monitoring and glucose management in T2DM individuals.

Individuals with type 2 diabetes mellitus (T2DM) face an elevated risk of depression compared to those without T2DM, with approximately one in every four T2DM individuals experiencing clinically significant depression.^1,2^ This comorbidity is concerning as depression has been linked to adverse outcomes, including an increased risk of cardiovascular diseases, heart failure and other health complications, ultimately diminishing the overall quality of life.^3^

A recent study highlighted the independent association between depression and impaired awareness of hypoglycaemia in type 1 diabetes.^4^ The reduction of hypoglycaemic symptoms and thus impaired counter-regulatory responses increased the risk of developing severe hypoglycaemia.^5^ Moreover, individuals with depression exhibit suboptimal concordance to treatment and dietary recommendations, leading to suboptimal glycaemic control and potentially placing them at a higher risk of hypoglycaemia.^6–8^ While hypoglycaemia has been observed commonly in individuals with diabetes, its prevalence and association with additional severe symptoms of depression underscore the need to explore whether individuals with depression are at a higher risk of hypoglycaemia compared to their counterparts without depression.

Furthermore, the dynamic nature of depressive symptoms in clinical practice adds another layer of complexity.^3^ Persistent and transient depression may exert distinct effects on the incidence of hypoglycaemia. Therefore, it is crucial for studies investigating the relationship between depression and hypoglycaemia to consider these variations. In light of these considerations, our investigation aims to explore the nuanced relationship between depression and the risk of hypoglycaemia among individuals with T2DM.

## Method

The ACCORD study, with 10 251 individuals (mean age 62 years and mean glycated haemoglobin (HbA1c) 8.3%), aimed to evaluate whether intensified management of blood glucose, blood pressure and lipid levels could enhance cardiovascular outcomes in individuals with T2DM. This study, with a median T2DM onset of ten years, had been detailed and published previously.^9–15^ However, because of the increased risk of cardiac death associated with intensive glycaemic control, the intervention was halted after an average follow-up of 3.7 years.^10^ Subsequently, all participants transitioned to standard glycaemic control, and the follow-up was continued. The ACCORD trial was funded by the National Heart, Lung, and Blood Institute (NHLBI), and its procedures were endorsed by both the NHLBI and local ethics committees, with all participants providing written consent after being fully informed about the trial. The data-sets generated and/or analysed during the current study are available in the BioLINCC repository (https://biolincc.nhlbi.nih.gov). There are no current plans to involve individuals in the dissemination of study findings.

The ACCORD Health-Related Quality of Life (HRQL) study, a subset of the ACCORD study, was specifically designed to prospectively evaluate the comprehensive impact of all ACCORD interventions on validated measures of depression and HRQL from the participant’s perspective. Within the ACCORD HRQL sub-study, depressive symptoms were systematically assessed using the nine-item Patient Health Questionnaire (PHQ-9), aligning with the DSM-IV^11^ criteria. Evaluations occurred at baseline and at 12, 36 and 48 months during clinical visits within the ACCORD study. The PHQ-9 comprised nine questions, each scored on a scale of 0–3 based on symptom severity. The severity of depressive symptoms was categorised as none (0–4 points), mild (5–9 points) or moderate–severe (10–24 points).^3,16^

The baseline characteristics of included participants, encompassing age, gender, race (White/Black and minority ethnic), duration of T2DM, living arrangements, education status, tobacco and alcohol consumption and medication usage, were collected through questionnaires, interviews and examination of medical records during recruitment. Smoking status was classified as never, former or current, while alcohol consumption was self-reported and quantified in terms of frequency per week. Key physiological parameters, including body mass index (BMI), systolic blood pressure (SBP), diastolic blood pressure (DBP) and heart rate, were measured by registered nurses at the assessment centre. Lipid profiles (total cholesterol, triglycerides, low-density lipoprotein cholesterol (LDL)), HbA1c and glomerular filtration rate (GFR) were assessed by the central laboratory, as detailed previously.^10,12,17^ The outcomes were hypoglycaemia requiring medical assistance (HMA) and hypoglycaemia requiring any assistance (HAA). During each visit, participants were queried about their encounters with ‘low blood sugar’. Severe hypoglycaemia was characterised by participants exhibiting a blood glucose level of <2.8 mmol/L or experiencing hypoglycaemic symptoms that promptly resolved with oral carbohydrates, intravenous glucose or parenteral glucagon. HMA was specifically defined as episodes necessitating admission to hospital or care by emergency response personnel.^18^ HAA was defined as hypoglycaemic episodes requiring assistance from a third party (e.g. family members or bystanders) without involvement of healthcare professionals. In this study, the analysis focused on the duration from randomisation until the occurrence of the first episode of HAA and/or HMA, in relation to the primary outcomes mentioned earlier.

Continuous variables were evaluated using analysis of variance or Mann–Whitney *U*-tests, while chi-square analysis based on distribution was employed for categorical variables. The association between depression and HMA or HAA was investigated through Cox proportional hazards regression. Depression was measured as a time-varying indicator at baseline, year 1 and year 3, both as categorical (none, mild or moderate–severe) and continuous (continuous PHQ-9 score) variables. Three analytical models were utilised: Model 1, which adjusted for age, race, gender, glucose control strategy, living arrangements, education status, duration of diabetes and cigarette and alcohol consumption; Model 2, which further adjusted for BMI, HbA1c and GFR, along with the variables in Model 1; Model 3, which additionally adjusted for hypoglycaemic drugs, including sulphonylurea, biguanide, meglitinide, alpha-glucosidase inhibitor, thiazolidinedione and regular insulin. We conducted further comparisons of hazard ratios between individuals who had ever had depression, persistent depression, persistent mild depression or persistent moderate–severe depression and those without a history of depression. Individuals without depression were utilised as the reference group. In addition, we compared the HbA1c levels to assess and visualise the effectiveness of blood glucose control among these groups. Subgroup and interaction analyses were performed based on age (≤60 years, >60 years), gender and blood control strategy (intensive or standard glucose control). All statistical analyses were two-sided, and significance was determined at *P* < 0.05. The analyses were carried out using Stata/MP software, version 17.0 for Windows (StataCorp LLC, College Station, TX, USA).

## Results

Out of the 10 251 individuals involved in the ACCORD study, 2053 were included in the HRQL sub-study. Some 1963 participants had baseline PHQ-9 data, 1871 had PHQ-9 data at 1 year, 1755 at 3 years and 1285 at 4 years (Table 1). Over a median follow-up period of 4.3 years (interquartile range: 3.8–5.2 years), 220 individuals (27.0 events per 1000 person-years) developed HAA, while 157 individuals (18.8 events per 1000 person-years) developed HMA. The baseline characteristics of the study participants are presented in Table 2. Participants with HAA or HMA tended to be older, had lower levels of education, higher BMI, a longer history of T2DM, were more likely to receive an intensive glucose control strategy and were more likely to receive sulphonylurea and insulin treatments.

**Table 1:** Participants meeting depression criteria at assessment points throughout the ACCORD trial

Visit	*N*	No depression, *n* (%)	Mild, *n* (%)	Moderate–severe, *n* (%)
Baseline	1953	1052 (53.9)	520 (26.6)	381 (19.5)
1 year	1860	1119 (60.1)	500 (26.9)	241 (13.0)
3 years	1753	1057 (60.3)	457 (26.1)	239 (13.6)
4 years	1282	774 (60.4)	344 (26.8)	164 (12.8)
Ever	–	1574	1107	620

**Table 2:** Baseline characteristics of participants based on hypoglycaemia status

	HAA (Yes)	HAA (No)	*P*-value	HMA (Yes)	HMA (No)	*P*-value
Variable	*N* = 220	*N* = 1733		*N* = 157	*N* = 1796	
PHQ-9	5.15 ± 5.10	5.35 ± 4.85	0.563	4.62 ± 4.86	5.39 ± 4.87	0.061
Age (years)	64.57 ± 6.84	62.58 ± 6.53	<0.001	65.50 ± 6.68	62.57 ± 6.54	<0.001
Duration of T2DM (years)	14.85 ± 8.48	10.55 ± 7.46	<0.001	15.35 ± 8.68	10.66 ± 7.50	<0.001
Gender, male	103 (46.82)	675 (38.95)	0.025	72 (45.86)	706 (39.31)	0.108
Race, White	132 (60.00)	1116 (64.40)	0.201	95 (60.51)	1153 (64.20)	0.356
Glucose control strategy			<0.001			<0.001
Standard	53 (24.09)	928 (53.55)		40 (25.48)	941 (52.39)	
Intensive	167 (75.91)	805 (46.45)		117 (74.52)	855 (47.61)	
Living alone	49 (23.27)	340 (19.79)	0.387	38 (24.20)	354 (19.71)	0.178
Education			<0.001			0.026
Less than high school	46 (20.91)	227 (13.11)		30 (19.11)	243 (13.55)	
High-school graduate	67 (30.45)	442 (25.53)		48 (30.57)	461 (25.70)	
Some college	65 (29.55)	575 (33.22)		50 (31.85)	590 (32.89)	
College degree or higher	42 (19.09)	487 (28.13)		29 (18.47)	500 (27.87)	
Current smoker	24 (10.91)	231 (13.33)	0.316	21 (13.38)	234 (13.03)	0.902
BMI (kg/m^2^)	31.72 ± 5.41	32.52 ± 5.39	0.040	31.69 ± 5.66	32.49 ± 5.36	0.076
HbA1c (%)	8.42 ± 0.97	8.26 ± 1.05	0.037	8.36 ± 0.85	8.27 ± 1.06	0.298
LDL (mg/dL)	104.73 ± 34.30	104.09 ± 33.69	0.792	101.97 ± 31.37	104.35 ± 33.95	0.397
Triglyceride (mg/dL)	162.47 ± 107.95	192.88 ±143.23	0.002	157.90 ± 108.22	192.20 ± 142.14	0.003
Total cholesterol (mg/dL)	182.35 ± 41.12	182.96 ± 41.03	0.837	179.31 ± 38.92	183.21 ± 41.21	0.254
SBP (mmHg)	137.92 ± 18.20	136.09 ± 16.89	0.134	138.52 ± 17.46	136.10 ± 17.00	0.089
DBP (mmHg)	72.44 ± 11.65	74.68 ± 10.78	0.004	71.79 ± 11.42	74.65 ± 10.83	0.002
Heart rate (bpm)	72.61 ± 12.62	72.41 ± 11.78	0.817	72.04 ± 12.24	72.47 ± 11.84	0.662
GFR (mL/min/1.73 m^2^)	86.58 ± 30.17	92.39 ± 31.23	0.009	84.46 ± 24.12	92.37 ± 31.63	0.002
Alcohol consumption per week (times)	0.70 ± 2.27	0.96 ± 2.65	0.177	0.66 ± 2.10	0.95 ± 2.65	0.183
Medication						
Sulphonylurea	98 (44.55)	946 (54.59)	0.005	68 (43.31)	976 (54.34)	0.008
Biguanide	132 (60.00)	1115 (64.34)	0.207	95 (60.51)	1152 (64.14)	0.364
Meglitinide	4 (1.82%)	36 (2.08)	0.798	2 (1.27)	38 (2.12)	0.475
Alpha-glucosidase inhibitor	4 (1.82%)	13 (0.75)	0.108	3 (1.91)	14 (0.78)	0.143
Thiazolidinedione	52 (23.64%)	404 (23.31)	0.915	35 (22.29)	421 (23.44)	0.744
Regular insulin	41 (18.64%)	186 (10.73)	<0.001	32 (20.38)	195 (10.86)	<0.001

Continuous variables are expressed as mean ± s.d. and categorical variables as *n* (%). HAA, hypoglycaemia requiring medical assistance; HMA, hypoglycaemia requiring any assistance; PHQ-9, nine-item Patient Health Questionnaire; T2DM, type 2 diabetes mellitus; BMI, body mass index; HbA1c, glycated haemoglobin; LDL, low-density lipoprotein cholesterol; DBP, diastolic blood pressure; SBP, systolic blood pressure; GFR, glomerular filtration rate.

**Fig. 1:**
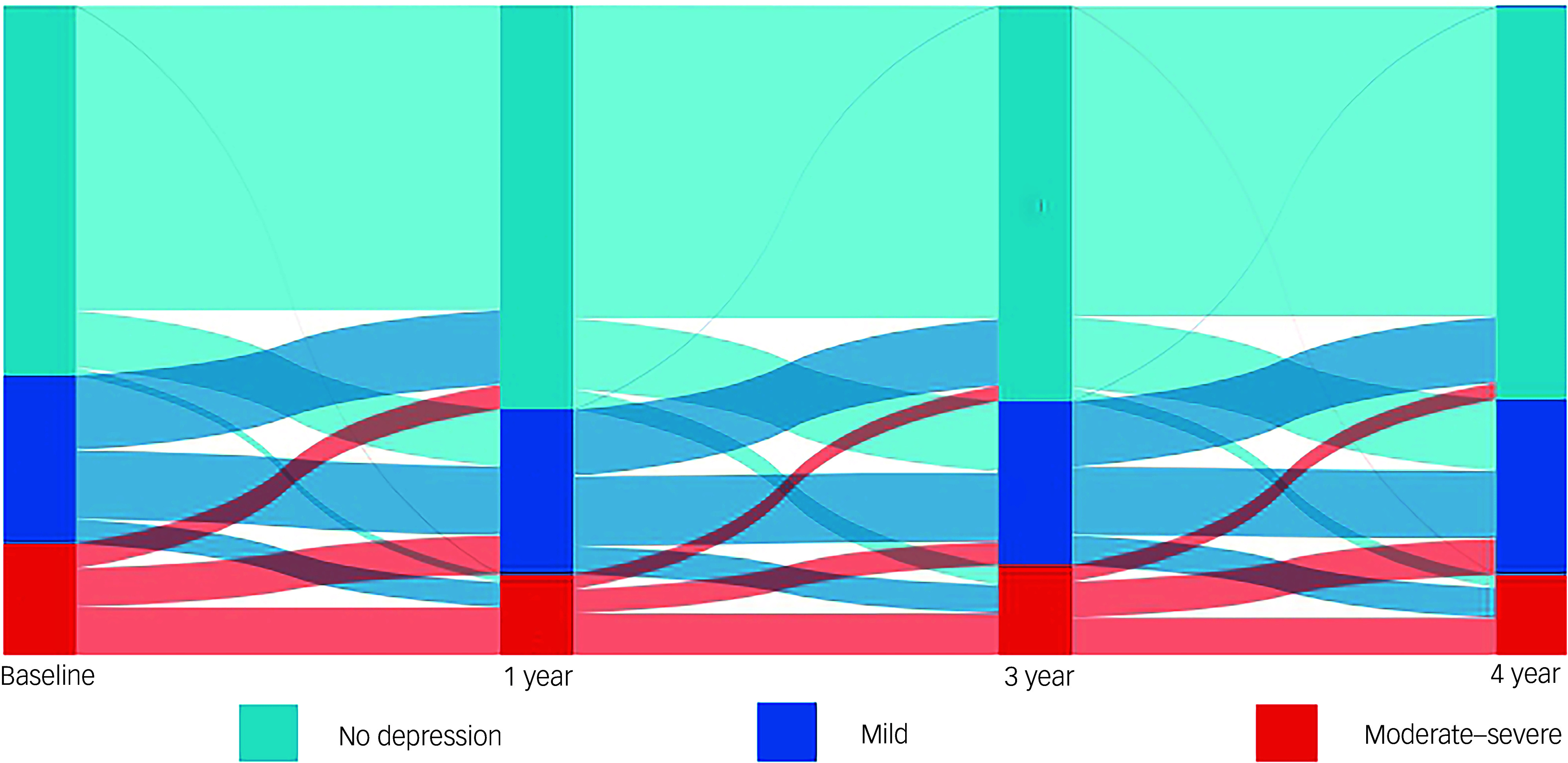
Depressive symptoms were evaluated at baseline and at 12, 36 and 48 months, illustrating their dynamic changes over time.

Table 1 illustrates the prevalence and incidence of mild or moderate–severe depression based on the PHQ-9 score at both baseline and during follow-up. At baseline, 520 participants (26.6%) exhibited mild depression, while 381 participants (19.5%) met criteria for moderate–severe depression. Figure 1 depicts longitudinal trends in depressive symptom trajectories among participants with complete follow-up data. There was a noticeable reduction in the prevalence of moderate–severe depression over the course of the follow-up duration. Depressive symptoms demonstrated dynamic changes during this period, with half of the participants who initially experienced moderate–severe depression reporting relief in their symptoms. Furthermore, a noteworthy proportion of participants, initially without depression or with mild depression, developed mild or moderate–severe depression, respectively. Nevertheless, participants without depression at the beginning of the study were less likely to progress to moderate–severe depression.

**Fig. 2:**
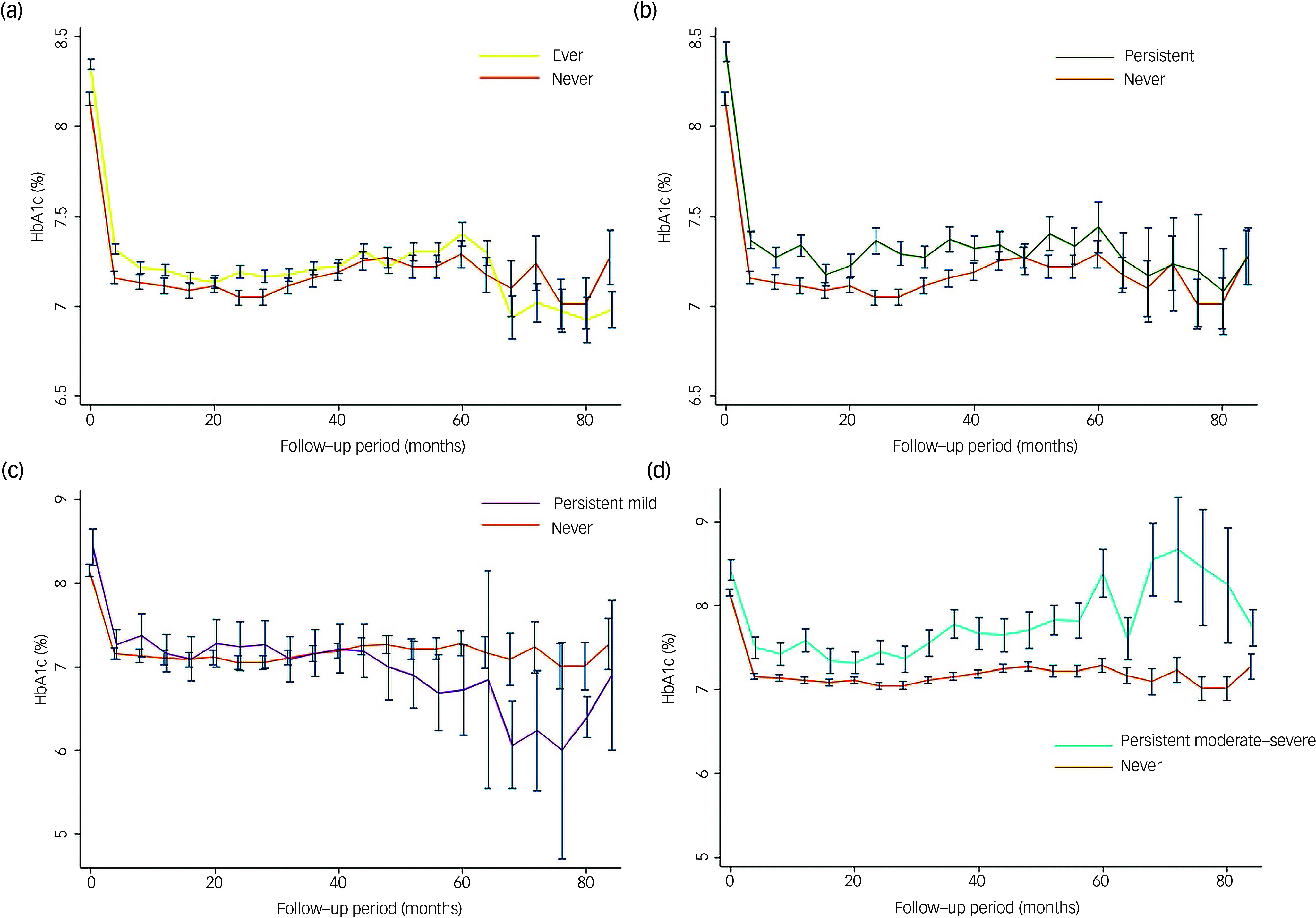
Comparison of glycated haemoglobin (HbA1c) levels and corresponding 95% confidence intervals between participants with and without depression: (a) ever had depression and never had depression; (b) persistent depression and never had depression; (c) persistent mild depression and never had depression; (d) persistent moderate–severe depression and never had depression.

Figure 2 illustrates the HbA1c levels and their respective 95% confidence intervals across different degrees of depression groups. Participants with varying degrees of depression exhibited less effective blood glucose control compared to their counterparts without depression. As the severity of depression increased, blood glucose control deteriorated. Notably, participants with persistent moderate–severe depression exhibited the least optimal blood glucose control. However, it is important to acknowledge a limitation in the analysis because of a low sample size in participants with persistent mild or moderate–severe depression over the 60-month period, primarily attributed to loss of follow-up. Consequently, the 95% confidence interval widens in Figs. 2(c) and 2(d), reflecting the impact of this limitation on the precision of the results in those specific groups.

Table 3 illustrates the association between the PHQ-9, both as a continuous variable and a categorical variable, and the incidence of HAA or HMA. According to Model 3, each unit increase in the PHQ-9 score did not show an associated increase in the risk of HAA or HMA (hazard ratio, 1.00, 95% CI, 0.97–1.03 for HAA; hazard ratio 0.98, 95% CI, 0.95–1.02 for HMA). Even after adjusting for relevant variables, individuals experiencing mild depression or moderate–severe depression did not exhibit a higher risk of HAA or HMA compared to those without depression. Similarly, participants with a history of ever experiencing depression or persistent depression (whether mild or moderate–severe) did not demonstrate a higher risk of HAA or HMA when compared to those who had never experienced depression. Subgroup analysis showed that age, gender and glucose control strategy did not moderate the association between depression measured by the PHQ-9 and HAA or HMA (Supplementary Table 1 available at https://doi.org/10.1192/bjo.2025.10853).

**Table 3:** Proportional hazard models of depression predicting hypoglycaemia events

	HAA (hazard ratio, 95% CI)			HMA (hazard ratio, 95% CI)		
	Model 1	Model 2	Model 3	Model 1	Model 2	Model 3
None	Ref.	Ref.	Ref.	Ref.	Ref.	Ref.
Mild^a^	0.93 (0.68,1.29)	0.94 (0.68,1.30)	0.91 (0.66,1.26)	0.95 (0.65,1.38)	0.93 (0.64,1.36)	0.90 (0.62,1.32)
Moderate–severe^a^	1.04 (0.70,1.53)	1.04 (0.70,1.53)	0.99 (0.66,1.47)	0.81 (0.49,1.34)	0.80 (0.48,1.32)	0.75 (0.45,1.26)
*P* for trend^a^	1.00	0.98	0.77	0.43	0.39	0.27
PHQ-9 continuous^a^	1.01 (0.98,1.04)	1.01 (0.98,1.04)	1.00 (0.97,1.03)	0.99 (0.95,1.03)	0.99 (0.95,1.03)	0.98 (0.95,1.02)
Never depression	Ref.	Ref.	Ref.	Ref.	Ref.	Ref.
Ever depression (*N* = 1912)^b^	0.89 (0.66,1.19)	0.89 (0.67,1.20)	0.87 (0.65,1.17)	0.81 (0.58,1.14)	0.81 (0.57,1.14)	0.78 (0.56,1.11)
Persistent depression (*N* = 1103)^b^	0.87 (0.59,1.30)	0.87 (0.59,1.30)	0.80 (0.53,1.20)	0.67 (0.41,1.09)	0.65 (0.40,1.07)	0.64 (0.40,1.07)
Persistent mild depression (*N* = 778)^b^	0.79 (0.39,1.61)	0.80 (0.39,1.63)	0.73 (0.36,1.51)	0.70 (0.30,1.66)	0.70 (0.30,1.67)	0.66 (0.28,1.58)
Persistent moderate–severe depression (*N* = 770)^b^	1.36 (0.74,2.50)	1.36 (0.74,2.50)	1.36 (0.74,2.50)	(0.81 (0.35,1.89)	0.80 (0.34,1.88)	0.71 (0.30,1.68)

Model 1: age, race, gender, glucose control strategy, living along, education status, duration of diabetes, cigarette and alcohol consumption.

Model 2: Model 1 in addition to body mass index (BMI), glycated hemoglobinhaemoglobin (HbA1c), glomerular filtration rate (GFR).

Model 3: Model 2 in addition to medication including sulphonylurea, biguanide, meglitinide, alpha-glucosidase inhibitor, thiazolidinedione, regular insulin.

HAA, hypoglycaemia requiring any assistance; HMA, hypoglycaemia requiring medical assistance; PHQ-9, nine-item Patient Health Questionnaire.

a Time-varying Cox.

b Cox.

## Discussion

This epidemiological analysis of data from the ACCORD-HRQL trial revealed the following in T2DM participants with high cardiovascular disease (CVD) risk: (a) depressive symptoms exhibited dynamic fluctuations during follow-up, emphasising the necessity for regular psychological monitoring and timely interventions; (b) depression at baseline or during the follow-up period was not associated with hypoglycaemia events but was associated with suboptimal glucose control, underscoring the critical role of optimised glucose management in this population.

The relationship between depression and glycaemic control remains controversial,^19–23^ with inconsistencies potentially attributable to methodological heterogeneity across studies. Divergent findings may stem from variations in depression assessment tools (e.g. the PHQ-9 versus the Center for Epidemiologic Studies Depression Scale (CES-D)), differences in study designs (cross-sectional versus longitudinal) and insufficient adjustment for confounders such as medication concordance. Persistent and transient depression may have different impacts on glycaemic control, contributing to conflicting findings. Previous studies primarily consisted of cross-sectional designs with relatively small sample sizes, resulting in weaker statistical power.^16–20^ Furthermore, the temporal variability of depressive symptoms – where persistent and transient states deferentially affect glucose regulation – introduces additional complexity.^24^ To address these limitations, this study employed a prospective cohort design to investigate the long-term association between depression severity and glycaemic control. By considering the dynamic changes in depression and blood sugar levels, the study conducted a total of 7187 depression assessments and 23 716 HbA1c measurements, resulting in robust statistical power. Our study found that depression may worsen glycaemic control by impeding effective diabetes self-management. In addition, Li et al observed that suboptimal glycaemic control could predict depression in individuals with T2DM.^25^ Therefore, further studies are needed to verify the bidirectional relationship.

Previous studies have found that hypoglycaemic events may trigger depression, but there is currently no research on whether depression can lead to hypoglycaemic events. For the first time, our study reveals that depression does not directly cause hypoglycaemic events in individuals with diabetes. Studies have suggested that depression may not directly influence blood glucose levels but rather affect the management of glycaemic control.^26^ Depressive symptoms may reduce engagement with treatment plans and dietary guidance, potentially contributing to elevated blood glucose levels.^23^ Depression is closely associated with reduced motivation, which significantly affects treatment concordance. Depressive symptoms often cause patients to disengage from treatment plans and ignore dietary guidance. As a result, instead of leading to hypoglycaemic events, this behavioural change potentially contributes to elevated blood glucose levels. While Heald et al reported a significant association between elevated HbA1c levels and hypoglycaemia-related admissions to hospital in older adults receiving insulin or insulin secretagogues,^24^ our cohort study failed to replicate this association after rigorous adjustment for antidiabetic medications. Our study uses data from the ACCORD trial, a randomised controlled trial (RCT) with a structured protocol, standardised interventions and close monitoring of participants. In contrast, Heald et al’s analysis draws on primary care databases reflecting real-world clinical practice, where patient management is more heterogeneous. Factors such as treatment adherence, follow-up frequency and intervention variability may differ substantially from those in a controlled trial setting, which could plausibly contribute to the observed differences in findings.

Our study holds significant clinical implications. Clinical physicians should regularly monitor the psychological status of individuals with T2DM, identify potential depression and intervene promptly, conducting regular assessments. We have observed that depression does not increase the risk of hypoglycaemia in individuals with T2DM; rather, it is associated with less effective glycaemic control. Therefore, prioritising glycaemic management is essential to improve the prognosis of individuals with type 2 diabetes and prevent diabetes complications. In real-world settings, where such intensive oversight may be less feasible, patients with depression may face greater challenges in maintaining optimal glucose control. Our findings regarding depression’s independent impact on diabetes outcomes may even underestimate the clinical relevance in routine care, as the combined effects of depression and suboptimal glucose control could further exacerbate adverse outcomes.

Our study has several limitations. First, there were a significant number of participants lost to follow-up for the PHQ-9 data in the fourth year. However, we found no significant differences in baseline characteristics between the lost to follow-up individuals and those with complete data. Second, it is important to acknowledge that the ACCORD study, being a RCT, had all participants’ blood glucose managed by healthcare professionals. This controlled setting may not fully reflect the real-world experiences of all individuals with T2DM. Third, while there is no specific behavioural or adherence data in the ACCORD data-set, the study protocol included regular supervision by healthcare professionals who adjusted medication dosages as needed. Finally, the exclusive recruitment from North America necessitates caution when extrapolating results to other ethnic or geographic populations.

Depressive symptoms fluctuate dynamically in individuals with T2DM, and those experiencing depression tended to exhibit less effective glycaemic control. However, there was no significant association between depression and increased hypoglycaemic events. These findings underscore the necessity for ongoing assessment and management of both mental health and glucose control in individuals with T2DM.

## Supporting information

Hong et al. supplementary material Hong et al. supplementary material

## Data Availability

The data-sets generated and/or analysed during the current study are available in the BioLINCC repository at https://biolincc.nhlbi.nih.gov

## Supplementary material

The supplementary material is available online at https://doi.org/10.1192/bjo.2025.10853

## Clinical trials registration numbers

Trial registration: http://www.clinicaltrials.gov. Unique identifier: NCT00000620.
