# Supplementary material for: Depressive symptoms and hypoglycaemic risk in individuals with type 2 diabetes mellitus: insights from the ACCORD-HRQL study
Source: BJPsych Open. 2025 Oct 13;11(6):e241. doi: 10.1192/bjo.2025.10853 (PMC12529320; doi:10.1192/bjo.2025.10853)
Supplement: Hong et al. supplementary material [file S2056472425108533sup001.pdf]

Supplementary Table 1: Subgroup analysis(continuous PHQ-9 and HAA or HMA)

		HAA#	P for interaction	HMA#	P for interaction
**Age**	**Age<60**	0.97(0.89,1.06)	0.39	0.95(0.88,1.03)	0.63
	**Age>=60**	0.99(0.93,1.04)		1.00(0.95,1.04)	
**Sex**	**Female**	0.99(0.94,1.03)	0.34	0.98(0.93,1.04)	0.93
	**Male**	1.02(0.97,1.07)		0.99(0.93,1.05)	
**Glucose control strategy**	**Standard**	0.97(0.91,1.04)	0.55	0.98(0.90,1.06)	0.91
	**Intensive**	1.01(0.97,1.05)		0.99(0.94,1.03)	

#Time varying Cox

Model 3: Model 2 in addition to medication including Sulphonylurea,Biguanide,Meglitinide Alpha-glucosidase inhibitor,Thiazolidinedione,Regular insulin,

HAA, Hypoglycemia requiring medical assistance; HMA, Hypoglycemia requiring any assistance;PHQ9,nine-item Patient Health Questionnaire;BMI,body mass index; HbA1c,glycosylated hemoglobin A1c;GFR, Glomerular filtration rate.
